# Licensed Dengue Vaccine: Public Health Conundrum and Scientific Challenge

**DOI:** 10.4269/ajtmh.16-0222

**Published:** 2016-10-05

**Authors:** Scott B. Halstead

**Affiliations:** 1 Department of Preventive Medicine and Biometrics, Uniformed Services University of the Health Sciences, Bethesda, Maryland

## Abstract

A tetravalent live attenuated vaccine composed of chimeras of yellow fever 17D and the four dengue viruses (chimeric yellow fever dengue [CYD]) manufactured by Sanofi Pasteur has completed phase III clinical testing in over 35,000 children 2–16 years of age. The vaccine was recently licensed in four countries. During the first 2 years of observation, CYD vaccine efficacy ranged between 30% and 79% in 10 different countries with an overall efficacy of 56.8%. During year 3, there was an overall efficacy against hospitalization of 16.7%, but a relative risk of hospitalization of 1.6 among children younger than 9 years and 4.95 in children 5 years of age and younger. Vaccination of seronegative children resulted in universal broad dengue neutralizing antibody responses, but poor protection against breakthrough dengue cases. Unless proven otherwise, such breakthrough cases in vaccinated subjects should be regarded as vaccine antibody-enhanced (ADE). The provenance of these cases can be studied serologically using original antigenic sin immune responses in convalescent sera. In conventional dengue vaccine efficacy clinical trials, persons vaccinated as seronegatives may be hospitalized with breakthrough ADE infections, whereas in the placebo group, dengue infection of monotypic immunes results in hospitalization. Vaccine efficacy trial design must identify dengue disease etiology by separately measuring efficacy in seronegatives and seropositives. The reason(s) why CYD vaccine failed to raise protective dengue virus immunity are unknown. To achieve a safe and protective dengue vaccine, careful studies of monotypic CYD vaccines in humans should precede field trials of tetravalent formulations.

## Introduction

A six-decade-long effort to develop a dengue vaccine culminated in December 2015, with the licensing of a tetravalent live attenuated yellow fever chimeric dengue vaccine in Brazil, El Salvador, Mexico, and the Philippines.^1^–^4^ This vaccine, Dengvaxia, is a mixture of chimeric yellow fever and dengue viruses (DENV) 1, 2, 3, and 4. Each component was developed by inserting the structural genes for the premembrane and envelope proteins of each of the four DENVs into the genes of the capsid and nonstructural proteins of yellow fever 17 D vaccine virus.^5^ After a decade of preclinical development at OraVax (Cambridge, MA), then Acambis Inc., in 2005, the company and dengue vaccine were acquired by Sanofi Pasteur, who managed further development and clinical testing. For the purposes of this presentation, this vaccine is designated chimeric yellow fever dengue (CYD).

## CYD Vaccine Efficacy Studies

Sanofi Pasteur conducted extensive phase III efficacy trials of their CYD vaccine, now in their third–fourth year, involving over 35,000 children, 2–16 years of age, resident in 10 dengue-endemic countries.^6^ During the first 2 years of observation, CYD vaccine efficacy against mild-severe dengue disease ranged between 30 and 79 in 10 different countries with an overall efficacy of 56.8%.^7^,^8^ During year 3, there was an overall efficacy against hospitalization of 16.7% (65 hospitalizations in vaccinees, 39 in placebo group), but a relative risk of hospitalization of 1.6 among children younger than 9 years and 4.95 in children 5 years of age and younger.^6^ Vaccination of seronegative children resulted in universal broad neutralizing antibody responses but poor protection against breakthrough dengue cases.^7^,^8^ During year 3, clinical observations on vaccinated children and placebo controls showed the vaccine to be asymmetrically protective and enhancing, that is, some age groups were protected, whereas in others, disease accompanying breakthrough dengue infections was increased. A review of published data suggests that “all or nearly all” hospitalizations of vaccinated children over the 3-year postvaccination period may have occurred in children who were susceptible when vaccinated, and are attributed to vaccine ADE.^9^

## Explanations of Efficacy Results

CYD developers have provided hypotheses to explain the third-year clinical responses observed during breakthrough DENV infections in vaccinated children.^10^ Summaries of these hypotheses receive comment here:

Hypothesis 1: *Antibody responses following administration of CYD wane more rapidly in younger than in older children, “consequently, their neutralizing responses are more likely to rapidly fall below protective thresholds for all four DENV serotypes and to present a monotypic pattern that is less likely to be cross-protective*.”^10^ Comment: The author knows of no evidence documenting differences in the kinetics of the dengue humoral responses of 2–5 year-old children compared with those in older children. Of importance in the context of dengue disease is the fact that CYD vaccine given to seronegatives regularly raised neutralizing antibodies, mostly dengue group specific.^11^–^13^ Despite these immune responses, in two trials, vaccination of seronegatives resulted in poor protection against subsequent mild and moderate DENV disease.^7^,^8^ The circulation of nonprotective DENV antibodies are established preconditions for antibody-dependent enhancement of DENV infection in Fc receptor-bearing cells (ADE) and animal models.^14^ The poor CYD protection observed in seronegative humans was presaged in preclinical testing. Administration of either monotypic DENV 2 or tetravalent CYD vaccines to susceptible monkeys raised DENV neutralizing antibodies, but these antibodies did not protect animals from developing anamnestic antibody responses after wild-type DENV challenge or occasional low-level viremia.^5^,^15^ It is true that enhanced viremia was not observed in vaccinated monkeys challenged 2–6 months after immunization. CYD vaccine may provide some degree of transient protection against enhanced disease similar to that described for wild-type DENV infections.^16^ Studies to identify the degree and nature of short-term heterotypic DENV protection afforded by CYD are urgently needed.

Hypothesis 2: *“Age differences at the microvascular and vascular levels could be associated with higher chances of plasma leakage, which is thought to contribute to severe disease.”*^10^ Comment: It is widely accepted that younger compared with older children or adults are intrinsically at greater risk of developing plasma leakage during a secondary DENV infection.^17^,^18^ The risk to ADE-mediated vascular permeability does not disappear at age nine. It is also important to recognize that the risk of developing plasma leakage during a second heterotypic DENV infection does not lessen, but increases with the length of the interval that a second DENV infection follows an initial DENV infection.^19^,^20^ CYD vaccine–induced antibody responses may carry the same risk.

Hypothesis 3: *Susceptibility (to severe disease) in vaccinated individuals is temporally clustered, therefore “a permanent predisposition to sensitization in seronegative vaccinated individuals is not compatible with the postulation behind this clustering hypothesis, and in particular such sensitization would no longer be present after a secondary-like infection has developed, in agreement with observations in the field showing a lower risk of developing severe disease on tertiary and quaternary infections.”*^10^ Comment: Exposure of a substantial portion of a population to infection with a single DENV during a limited period of time is a common feature of dengue epidemiology. For example, in 1977–1979, Cuba experienced an island-wide DENV 1 infection. In 1981, enhanced DENV-2 disease occurred in cohorts who had been infected with DENV-1.^21^ The 1977–1979 DENV-1-immune cohort (or cluster) experienced enhanced disease response to breakthrough DENV-2 and DENV-3 infections more than 20 years later.^22^,^23^ It is likely that individuals vaccinated in a cluster will have the following responses: seronegative individuals receiving vaccine may experience a short-term, incomplete protection against wild-type DENV infections and disease. Within a year or two, this protection may wane permitting a subsequent DENV infection (first DENV infection for the individual) any time thereafter to produce enhanced infection and disease. This breakthrough DENV infection elicits a secondary immune response. Seropositives have their immune response broadened by vaccine preventing further DENV infection and disease. The actual post-CYD vaccination scenario concerning DENV infection and disease of individuals vaccinated when susceptible requires further study over an extended period.

## Why do Yellow Fever Dengue Chimeric Viruses Fail to Protect Against Dengue Infection?

The empiric nature of dengue vaccine development and the requirement for individual testing of each monovalent element, including, critically, demonstration of protection using a human challenge model has received prior discussion.^24^ Since that review, important new understanding has been gained concerning the structure and function of monovalent DENV neutralizing antibodies.^25^ But, does tetravalent CYD vaccination raise these “protective” monovalent antibodies? Evidence of a protective role of T-cells in human DENV infections has been mounting.^26^ T-cells directed at nonstructural protein epitopes similar to those observed in individuals infected with wild-type DENV have been detected in recipients of the live attenuated tetravalent dengue vaccine produced by the National Institutes of Health.^27^ Might T-cell immunity after administration of CYD vaccine be blunted because yellow fever not DENV nonstructural proteins are presented? Finally, must an effective dengue vaccine contain dengue NS1? An analogy has been observed between the function in humans of bacterial lipopolysaccharides (LPSs) and that of DENV NS1.^28^ Both compounds interact with toll-like receptor 4 (TLR4) on the surface of monocytes, macrophages, and endothelial cells to induce the release of a range of cytokines and chemokines. These same mediators have been identified in the blood of patients with dengue hemorrhagic fever (DHF)/dengue shock syndrome (DSS). NS1-mediated cytokine release was inhibited by the TLR4 antagonist LPS-*Rhodobacter sphaeroides* suggesting an avenue for therapeutic intervention. Crucially, this same observation has been confirmed in an in vivo model. DENV 2 NS1 inoculated intravenously at physiologically relevant concentrations in sublethal DENV 2-infected interferon-α/β receptor −/−C57BL/6 mice produced lethal vascular permeability.^29^ In vitro, NS1 when added to cultured human endothelial cells resulted in endothelial permeability and disruption of endothelial cell monolayer integrity. These observations suggest that DSS may be a viral protein toxicosis. It was further shown that vaccination of mice with DENV 2 NS1 protected against endothelial leakage and death due to lethal DENV 2 challenge. Immunization with DENV 1, 3, and 4 NS1 proteins partially protected against heterologous DENV 2 challenge. The successful prevention of death in mice due to DENV by immunizing with NS1 was established long ago and repeated many times.^30^,^31^ Is it possible that the CYD fails to protect seronegatives because it does not contain DENV NS1 antigens?

Because published data document that CYD vaccine protected 74–84% of seropositives against mild-moderate disease, it is possible that classical primary or secondary CYD vaccine failure occurred. Who are the “seropositives” who were not protected? Might these individuals have experienced prior infections/immunizations with a non-DENV flavivirus? This important question requires more study. Breakthrough clinical cases can be studied retrospectively to identify sequential infections serologically. Several approaches are available: 1) test early convalescent sera for enzyme-linked immunosorbent assay (ELISA) yellow fever (YF) or DENV NS1 IgG or IgM antibodies.^32^ The presence of IgG DENV NS1 antibodies signifies the patient is experiencing a secondary DENV infection (primary vaccine failure), whereas the absence of these antibodies and the presence of YF IgG NS1 or DENV IgM NS1 antibodies suggests that the CYD vaccine provided the sensitizing infection. 2) Test early convalescent sera for ELISA antibodies to DENV 1–4 domain III antigens.^33^ The identity of first DENV infection is revealed via the phenomenon of original antigenic sin. It is also possible that CYD vaccination of susceptibles raises DENV 4 antibodies that sensitize to ADE.^13^,^34^

## Impact of CYD Efficacy Trial Outcomes on Future Dengue Vaccine Efficacy Design

The CYD tetravalent vaccine is closely followed in clinical testing by two additional live attenuated tetravalent dengue vaccines, each containing chimeric viruses; the Takeda vaccine contains chimeras of DENV-1, 3, and 4 on a DENV-2 backbone,^35^ whereas the tetravalent National Institute of Allergy and Infectious Diseases vaccine contains a DENV-2/4 chimera.^36^ To assure that these chimeras are fully protective, the efficacy trial design must be changed.

Although guidelines for CYD dengue vaccine efficacy studies were drawn-up by international experts, they did not correctly anticipate CYD phase III trial outcomes.^37^–^39^ World Health Organization (WHO) experts recognized that “a subimmunogenic vaccine, or a vaccine whose efficacy wanes over time, could leave a recipient with an ‘immune profile’ which not only fails to protect, but increases the risk for experiencing severe dengue through complex immunopathological mechanisms following subsequent natural infection.” The guidelines also concluded that “protection can be measured only if vaccinated and control subjects are equally at risk to mild and severe dengue.” Despite this warning, the WHO did not identify the possibility that susceptibles, when vaccinated, would be converted to monotypic DENV-immune equivalents “at risk” to enhanced disease accompanying breakthrough DENV infections. In CYD vaccine trials, vaccinated and control subjects were not equally at risk to disease. In the vaccine cohort, DENV disease of all degrees of severity was likely due to infection of individuals who had been vaccinated as seronegatives. In the placebo group, DENV infection of seronegatives results only in mild disease, whereas a majority of moderate and hospitalized dengue cases occur in DENV-infected monotypic immunes (seropositives). DENV disease etiology differs in the two groups. The populations are “apples” and “oranges.”

The correct design to estimate efficacy requires that DENV disease responses be adequately measured for seronegatives and seropositives in the vaccinated and placebo groups separately. This requires that all or a substantial portion of enrolled phase III clinical trial participants be bled before administration of vaccine or placebo and at yearly intervals thereafter. Should there be no DENV disease of any degree of severity in vaccinated seronegatives, vaccine efficacy may be judged to be 100%. This same calculation using imagined data should be made for the seropositive group. Efficacy and relative risk may be calculated as illustrated in Table 1, in this instance for hospitalized disease. In this example, children, 2–16 years of age, 35% of who are seronegative were vaccinated and exposed during year 3 to a DENV infection rate of 16%. If, among the 560 DENV infections that occurred, 10 were hospitalized and in the placebo group, among 280 DENV infections, one child was hospitalized, the vaccine conveys a relative risk of 5.0. If calculated for mild/moderate disease, especially during the first 2 years after vaccination, positive vaccine efficacy may be identified. For seropositives, it is assumed that 37.8% of 10,000 vaccinees are monotypic immunes, and at a 16% DENV infection rate, 605 are infected, yielding two hospitalizations. Among 302 DENV-infected placebo monotypic DENV-immunes, 10 were hospitalized for an efficacy of 90%.

## Identifying Vaccine-Enhanced Dengue Disease

Clearly, any dengue infection occurring in a subject receiving a dengue vaccine is evidence of vaccine failure. Clinically, however, the mild disease that accompanies primary dengue infections in children may exactly mimic dengue disease occurring in the presence of antibodies raised by the vaccine. Vaccination may raise mixtures of protective and enhancing antibodies, and with the passage of time, this balance may shift toward enhancement. Categorizing mild disease as “vaccine-enhanced” requires evidence of a statistically significant increased rate of such disease among vaccinated compared with appropriate placebo controls. Because hospitalized disease, DHF, or severe dengue seldom accompany primary dengue infections in children, when such cases occur among vaccinated individuals, they should be recognized as serious adverse events, that is, vaccine-enhanced dengue disease.

## Can the CYD Vaccine be Used Safely to Control Dengue Disease?

On the basis of the described protection of seropositives and reduction of severe disease in the CYD phase III clinical trials, it is expected that one or more doses of vaccine will efficiently protect the high-risk group—monotypic DENV-immunes—from acquiring disease when exposed to DENV.^6^ The duration of this protective immunity is unknown. Neither is it known whether vaccination of monotypic immunes prevents subsequent DENV infection and viremia. Today worldwide, DENV infections of susceptible and monotypic-immune adults contribute to the pool of virus in circulation. According to Sanofi, CYD will not be given to children under the age of 9 years. Children in this age group should sustain community DENV force of infection at fairly high rates well into the future even in the face of massive vaccination of individuals 9 years of age and older. High rates of immunization of individuals 9 years of age and older can be expected to reduce illness burden substantially, but only among monotypic immunes. In Mexico, where a large percent of vaccinated population is seronegative (∼47%), overall protective efficacy was low (31.3%).^40^ The low efficacy must be the result of high rates of vaccine-enhanced DENV disease. The challenge confronting public health officials is how to immunize those who will benefit from vaccination but shield those who are at risk to vaccine-acquired enhanced DENV disease. To do this, it has been suggested that the vaccine be given only to dengue immunes.^9^ Manufacturers, regulators, and public health authorities must grapple with this question to find an equitable, affordable, and ethical solution.

## Figures and Tables

**Table 1:** Model with hypothetical data for estimating efficacy and relative risk in children 2–16 years of age, using a dengue vaccine that may sensitize vaccinated seronegatives to hospitalized DENV disease (Three years after initial vaccination)

	Vaccinated population (*N* = 10,000)	Placebo population (*N* = 5,000)
Seronegatives: direct calculation		
% Seronegative	35	35
Seronegative	3,500	1,750
DENV infection rate (%)	16	16
DENV 1–4 infections	560	280
Hospitalized	10*	1*
Hospitalization rate	10/560 (1.79%)	1/280 (0.4%)
Relative risk = 5.0, *P* = 0.1		
Seropositives: classical calculation		
% Monotypic immunes	37.8%	37.8%
Monotypic immunes	3,780	1,890
DENV infection rate	16%	16%
	Exposed to DENV: 605	2nd DENV infections: 302
Hospitalized	2*	10*
Vaccine efficacy: 90%		

DENV = dengue virus.

* These cases must be characterized serologically. If hospitalizations in vaccinated seronegatives show attributes of a yellow fever-dengue chimeric–DENV infection, case is vaccine-related ADE. In vaccinated seropositives (if they occur), cases that show evidence of secondary DENV infection identify vaccine failure.
